# Metformin potentiates the effect of arsenic trioxide suppressing intrahepatic cholangiocarcinoma: roles of p38 MAPK, ERK3, and mTORC1

**DOI:** 10.1186/s13045-017-0424-0

**Published:** 2017-02-28

**Authors:** Sunbin Ling, Haiyang Xie, Fan Yang, Qiaonan Shan, Haojiang Dai, Jianyong Zhuo, Xuyong Wei, Penghong Song, Lin Zhou, Xiao Xu, Shusen Zheng

**Affiliations:** 1 Division of Hepatobiliary and Pancreatic Surgery, Department of Surgery, First Affiliated Hospital, School of Medicine,, Zhejiang University, Hangzhou, China; 2 Key Laboratory of Combined Multi-organ Transplantation, Ministry of Public Health, Hangzhou, China; 3 Key Laboratory of Organ Transplantation, Hangzhou, Zhejiang Province China; 4 Collaborative Innovation Center for Diagnosis and Treatment of Infectious Diseases, Hangzhou, China

**Keywords:** Metformin, Arsenic trioxide, Intrahepatic cholangiocarcinoma, mTORC1, p38 MAPK, ERK3

## Abstract

**Background:**

Arsenic trioxide (ATO) is commonly used in the treatment of acute promyelocytic leukemia (APL), but does not benefit patients with solid tumors. When combined with other agents or radiation, ATO showed treatment benefits with manageable toxicity. Previously, we reported that metformin amplified the inhibitory effect of ATO on intrahepatic cholangiocarcinoma (ICC) cells more significantly than other agents. Here, we investigated the chemotherapeutic sensitization effect of metformin in ATO-based treatment in ICC in vitro and in vivo and explored the underlying mechanisms.

**Methods:**

ICC cell lines (CCLP-1, RBE, and HCCC-9810) were treated with metformin and/or ATO; the anti-proliferation effect was evaluated by cell viability, cell apoptosis, cell cycle, and intracellular-reactive oxygen species (ROS) assays. The in vivo efficacy was determined in nude mice with CCLP-1 xenografts. The active status of AMPK/p38 MAPK and mTORC1 pathways was detected by western blot. In addition, an antibody array was used screening more than 200 molecules clustered in 12 cancer-related pathways in CCLP-1 cells treated with metformin and/or ATO. Methods of genetic modulation and pharmacology were further used to demonstrate the relationship of the molecule. Seventy-three tumor samples from ICC patients were used to detect the expression of ERK3 by immunohistochemistry. The correlation between ERK3 and the clinical information of ICC patients were further analyzed.

**Results:**

Metformin and ATO synergistically inhibited proliferation of ICC cells by promoting cell apoptosis, inducing G0/G1 cell cycle arrest, and increasing intracellular ROS. Combined treatment with metformin and ATO efficiently reduced ICC growth in an ICC xenograft model. Mechanistically, the antibody array revealed that ERK3 exhibited the highest variation in CCLP-1 cells after treatment with metformin and ATO. Results of western blot confirm that metformin and ATO cooperated to inhibit mTORC1, activate AMP-activated protein kinase (AMPK), and upregulate ERK3. Metformin abrogated the activation of p38 MAPK induced by ATO, and this activity was partially dependent on AMPK activation. Inactivation of p38 MAPK by SB203580 or specific short interfering RNA (siRNA) promoted the inactivation of mTORC1 in ICC cells treated with metformin and ATO. Activation of p38 MAPK may be responsible for resistance to ATO in ICC. The relationship between p38 MAPK and ERK3 was not defined by our findings. Finally, AMPK is a newfound positive regulator of ERK3. Overexpression of EKR3 in ICC cells inhibited cell proliferation through inactivation of mTORC1. ERK3 expression is associated with a better prognosis in ICC patients.

**Conclusions:**

Metformin sensitizes arsenic trioxide to suppress intrahepatic cholangiocarcinoma via the regulation of AMPK/p38 MAPK-ERK3/mTORC1 pathways. ERK3 is a newfound potential prognostic predictor and a tumor suppressor in ICC.

**Electronic supplementary material:**

The online version of this article (doi:10.1186/s13045-017-0424-0) contains supplementary material, which is available to authorized users.

## Background

Cholangiocarcinoma is categorized as an intrahepatic, perihilar, and distal cholangiocarcinoma that is highly lethal [1]. Intrahepatic cholangiocarcinoma (ICC) is the second most common type of primary liver cancer [2], and in recent decades, the incidence and mortality rates for this cancer have been increasing worldwide. Although treatment approaches, such as surgical resection, liver transplantation, systemic chemotherapy, transarterial chemoembolization (TACE), and radiofrequency ablation, were developed as therapeutics for ICC, the prognosis of ICC patients is still very poor. For advanced stage disease, systemic pharmacotherapy is usually the primary treatment. Cisplatin plus gemcitabine is the first option for treatment of patients with advanced biliary cancer, but this treatment achieves a median overall survival of only 11.7 months [3].

Metformin, an anti-type II diabetes agent widely used throughout the world, has been recognized as a potentially preventive and therapeutic anticancer drug, according to recent epidemiological surveys and laboratory studies. In 2013, an epidemiological study that included 1828 potential ICC patients reported that, in diabetic patients, metformin use was associated with a 60% reduction in ICC risk [4]. However, in 2015, the same research group from the Mayo Clinic analyzed 250 patients with cholangiocarcinoma and observed that metformin could not improve the survival of cholangiocarcinoma patients with diabetes mellitus [5]. We previously reported that metformin could inhibit the growth of ICC cells in vitro [6], and another excellent study displayed similar results [7]. Moreover, we further found that metformin effectively sensitized ICC cells to certain chemotherapeutic agents, such as sorafenib, 5-fluorouracil, and arsenic trioxide (ATO). Thus, as treatment with metformin alone seems ineffective for ICC, combination of metformin with conventional chemotherapeutics may elevate the therapeutic effect. For example, some recent studies have revealed positive results using metformin as chemosensitizer or radiosensitizer [8, 9].

ATO is a traditional Chinese medicine that is mainly used to treat acute promyelocytic leukemia (APL). For solid tumors, although numerous studies have demonstrated the antitumor activity of ATO in vitro and in vivo [10, 11], treatment with ATO alone did not benefit patients [12]. Moreover, the required doses of ATO increased the risk of side effects and seriously limited further clinical use of ATO. Interestingly, when combined with other agents or radiation, ATO showed treatment benefits with manageable toxicity [12]. High dose and drug resistance might mediate the failure of single ATO treatment in solid tumor [12]. Accordingly, new strategies are needed to enhance the antitumor activity of ATO or reverse the drug resistance while reducing the required dose and ATO-associated side effects.

In ICC cells, we found that metformin amplified the inhibitory effect of ATO more significantly than other agents. Our previous study reported that metformin could enhance the antiproliferative effects of ATO in hepatocellular carcinoma (HCC) cells [13]. In addition, numerous studies have shown that the combination of ATO with certain agents exhibited synergistic anticancer effects against solid tumors [14–16]. Thus, we designed this study to investigate the chemotherapeutic sensitization effect of metformin in ATO-based treatment in ICC in vitro and in vivo, and we further explored the underlying mechanism.

## Results

### Metformin and ATO synergistically inhibited proliferation of ICC cells by promoting cell apoptosis, inducing G0/G1 cell cycle arrest, and increasing intracellular ROS

**Fig. 1:**
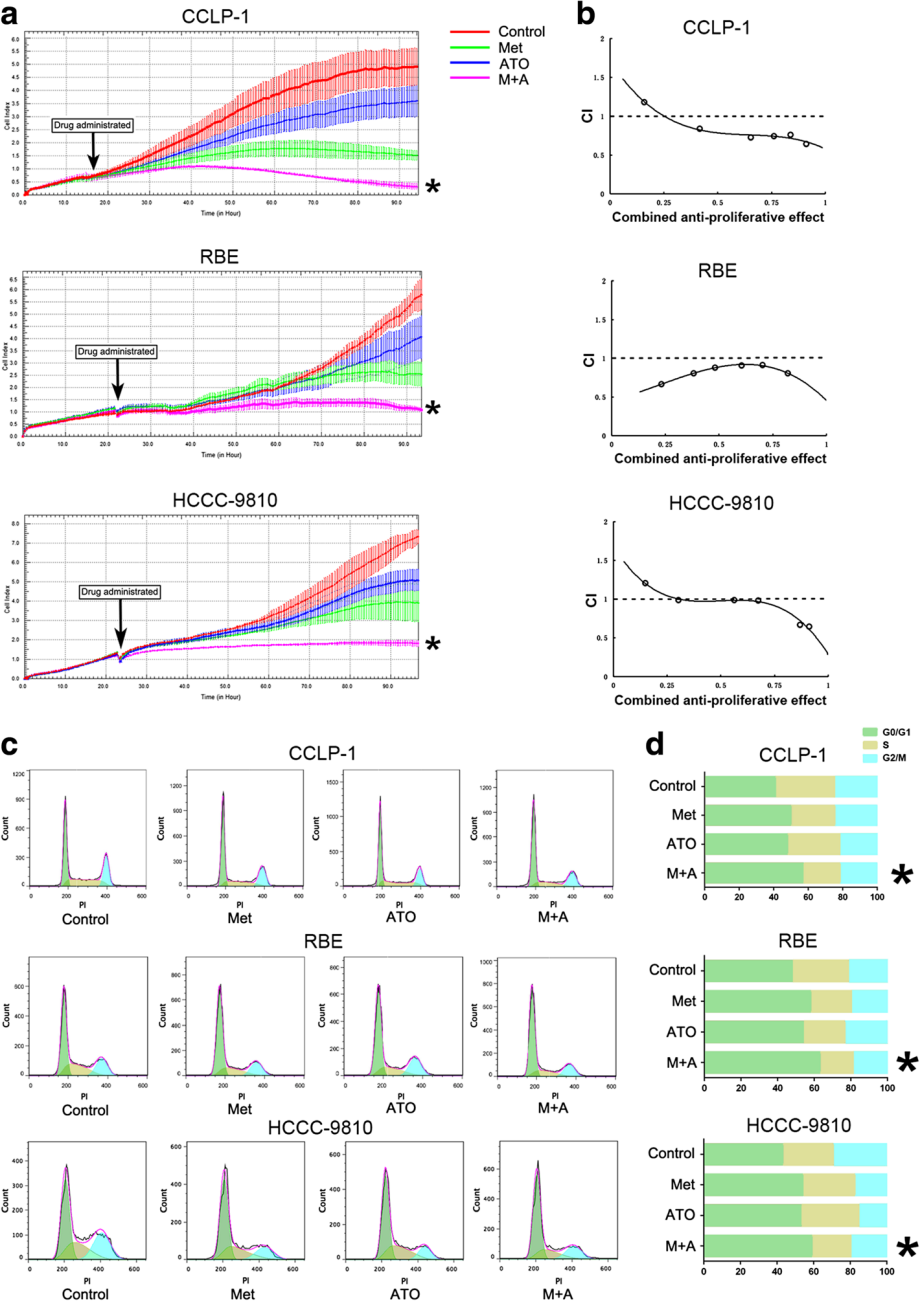
Combination treatment with metformin and ATO synergistically suppresses proliferation in ICC cells in vitro. **a** After treatment with 10 mmol/L metformin and 3 μmol/L ATO in combination or single treatments in ICC cells, the real-time cell growth was monitored for 72 h by using RTCA Analyzer and the growth cures were shown. **b** ICC cells were treated with different concentrations of metformin and ATO for 48 h, CCK assay was used evaluating the cell viability. The combination index was calculated as described in Methods. CI values less than one is considered synergism. **c** and **d** After 48 h of treatment with 10 mmol/L metformin, 3 μmol/L ATO, or their combination, ICC cells were examined using PI staining and the cell cycle distribution was measured by flow cytometric analysis. (Combination vs metformin or combination vs ATO ^*^ *P* < 0.05)

The antiproliferative effects of combination treatment with metformin and ATO were investigated in CCLP-1, RBE, and HCCC-9810 cells using a system of real-time cell growth monitoring with an RTCA DP Analyzer. As shown in Fig. 1a, metformin (10 mM) in combination with ATO (3 μM) nearly abrogated the growth of ICC cells, according to the cell index. Moreover, to investigate whether metformin plus ATO acts synergistically, a median effect analysis [17] was performed, which showed that the combination index (CI) value trended toward values of less than one and then further decreased, as the combined antiproliferative effects increased, indicating the combination was synergistic (Fig. 1b). The concentrations of the two agents in each group and the corresponding combined antiproliferative effect values and Cis are listed in Additional file 1.

**Fig. 2:**
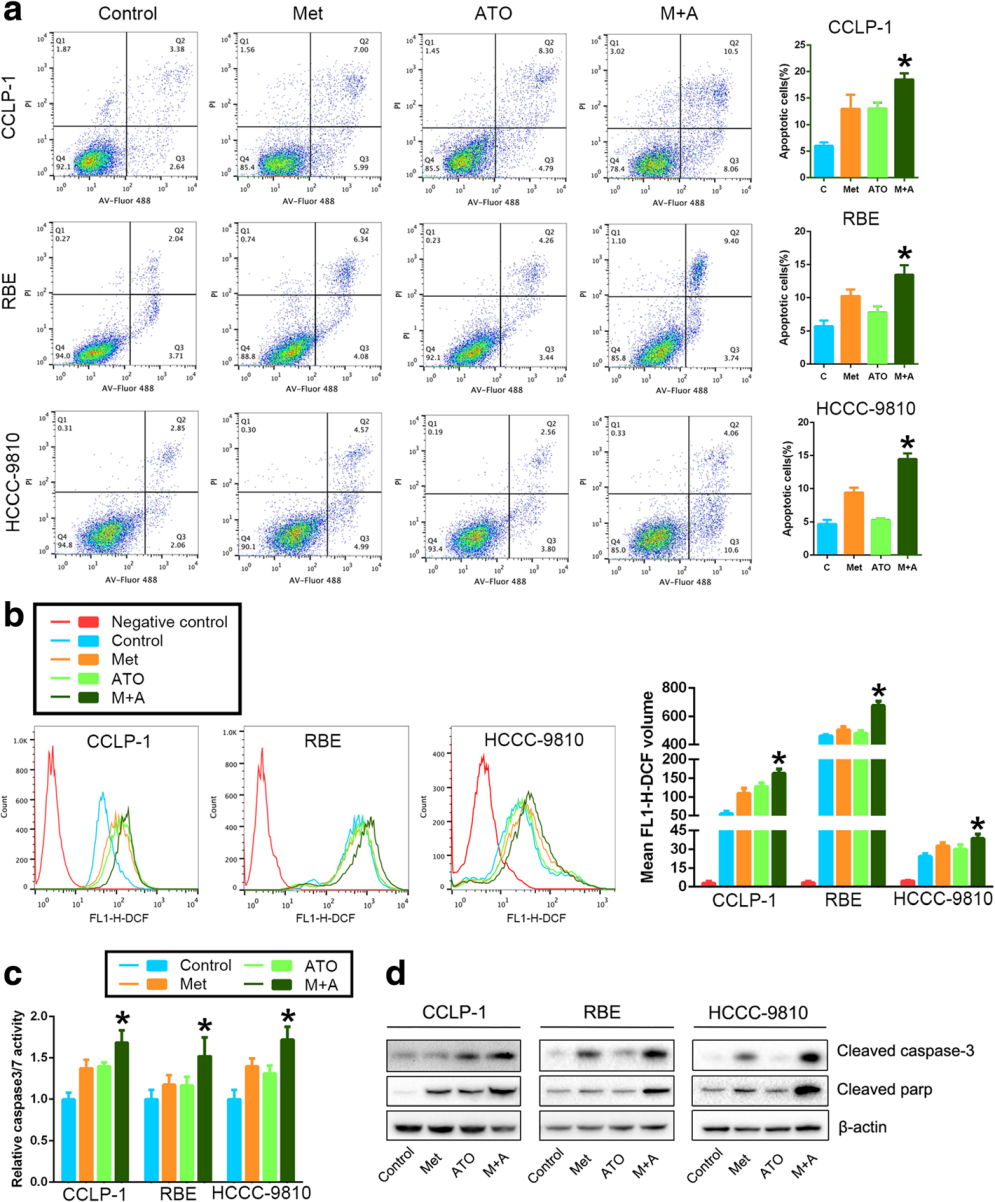
Metformin facilitates the pro-apoptotic effects of ATO on ICC cells through increasing the ROS production induced by ATO. **a** After treatment with 10 mmol/L metformin, 3 μmol/L ATO, or a combination of both, ICC cells were examined using Annexin V/PI staining, and the distribution of apoptotic cells was measured by flow cytometry. The percentages of early apoptotic cells plus late apoptotic/necrotic cells are shown in the bar graph. **b** Cells were treated with 10 mmol/L metformin, 3 μmol/L ATO, or a combination of both for 24 h. The intracellular ROS were measured by flow cytometric analysis using an oxidation-sensitive fluorescent probe, DCFH-DA, which is oxidized to DCF by ROS (the negative control was not treated with DCFH-DA). The mean volumes of DCF are shown in the bar graph as the means ± SD from three independent experiments. **c** Caspase3/7 activity in ICC cells treated with 10 mmol/L metformin, 3 μmol/L ATO, or a combination of both. **d** Cleaved caspase-3 and cleaved PARP were monitored using western blot analysis. (Combination vs metformin or combination vs ATO ^*^ *P* < 0.05)

Next, we performed a cell cycle and apoptosis analysis. A significant increase in the number of cells arrested in G0/G1 phase was observed in ICC cells treated with a combination of metformin and ATO compared to the single-drug treatments, as shown in Fig. 1c, d. Similarly, the apoptosis assay, as shown in Fig. 2a, revealed that a significant increase in the number of apoptotic cells was observed in ICC cells with a combination of metformin and ATO treatment, compared to the single-drug treatment. Consistent with the greater apoptotic events, the combined treatment led to higher levels of cleaved PARP and cleaved Caspase-3 and Caspase-3/7 activity in the three ICC cell lines compared to the single-drug treatment alone (Fig. 2c, d). As ATO can induce mitochondrial-dependent ROS production in cancer cells [18], we further determined the influence of metformin on ATO-induced ROS production. As expected, the data in Fig. 2b shows that metformin (10 mM) readily promoted ATO (3 μM)-induced ROS production.

Collectively, these results revealed that combined treatment with metformin and ATO remarkably promotes antiproliferative effects by promoting cell apoptosis, inducing G0/G1 cell cycle arrest, and increasing intracellular ROS.

### Metformin and ATO regulate mTORC1, AMPK/MAPK, and ERK3 in ICC cells in vitro

**Fig. 3:**
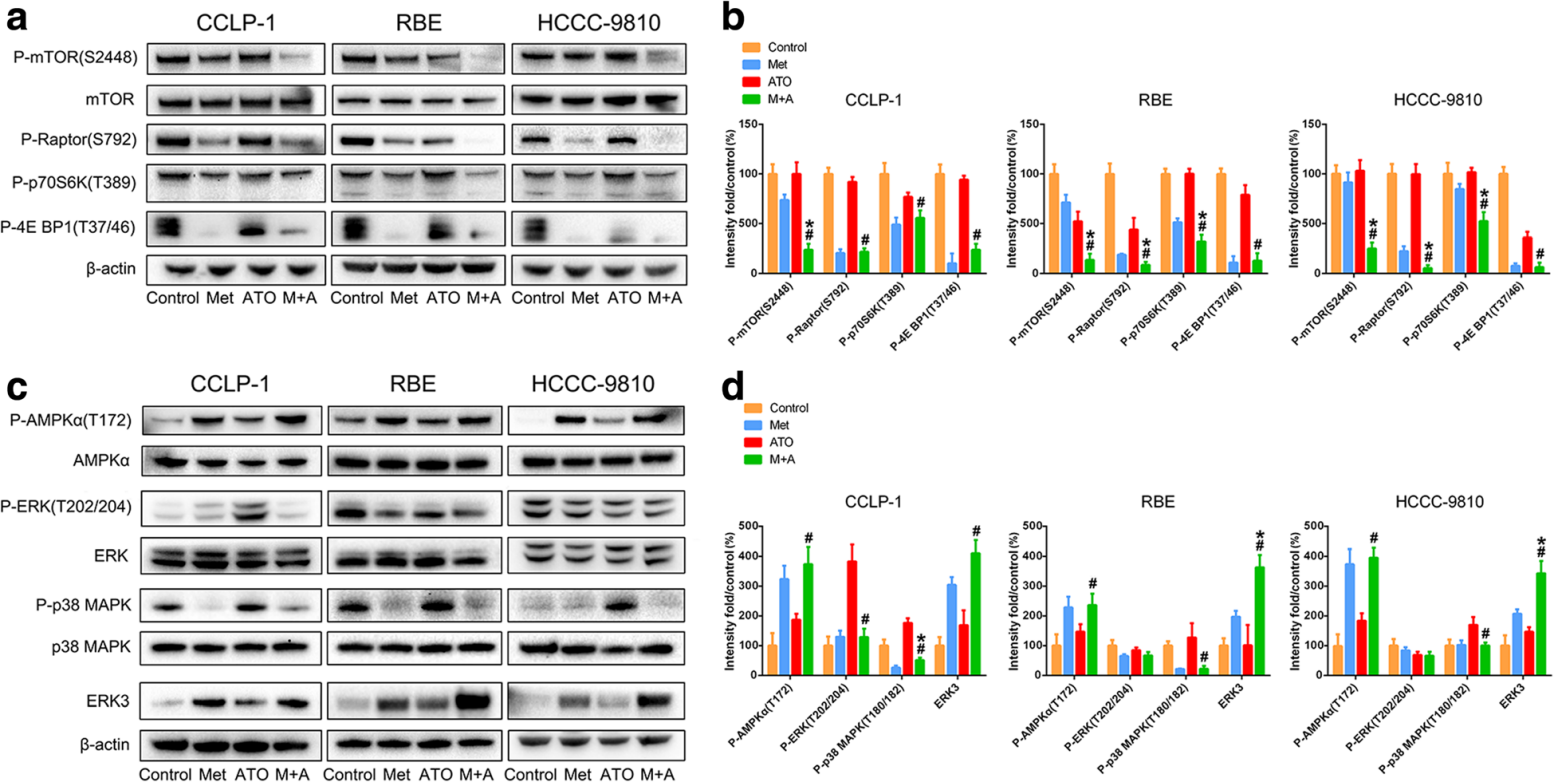
The effects of metformin and ATO on the mTORC1 and AMPK/MAPK pathways in ICC in vitro. **a** and **c** The effect of metformin and ATO single or combination treatment on the active status of mTORC1, AMPK/MAPK, and ERK3 pathways of ICC cells was determined by western blotting. **b** and **d** The data were quantified and are represented as the means ± SD from three independent experiments. (Combination vs metformin ^*^ *P* < 0.05, combination vs ATO ^#^ *P* < 0.05)

To analyze the potential molecular mechanism, we investigated the effects of metformin and ATO on the mTORC1 and AMPK/MAPK pathways in ICC cells. Metformin and ATO cooperated to abrogate the activation of mTORC1 (p-mTOR Ser2448, p-p70S6K Thr389, p-Raptor Ser792, p-4E BP1 Thr37/46), as shown in Fig. 3a, b. The inactivation effect of metformin (10 mM) on mTORC1 seemed to be more profound than that of ATO (3 μM). The consequences of combination treatment with metformin and ATO on AMPK/MAPK networks were further investigated (Fig. 3c, d), revealing that metformin and ATO could activate AMP-activated protein kinase (AMPK) while the p-ERK (T202/204) regulated by metformin, and ATO was inconsistent in the three cell lines used. However, activation of p38 MAPK was observed in ATO-treated cells, and this activation appeared to be almost completely abrogated by metformin.

Moreover, to explore the molecular mechanism in detail, we used a phospho-antibody array and screened more than 200 molecules clustered in twelve cancer-related pathways and found that total ERK3 exhibited the highest variation in ICC cells after treatment with metformin and ATO. The array data were quantified and are listed in Additional file 2. ERK3, encoded by MAPK6, is an atypical member of the MAPK family and is, thus far, primarily believed to be an antitumor molecule [19, 20]. Although, recent studies have reported that ERK3 can promote cancer cell migration and invasion [21, 22]. Thus, to further define the role of EKR3 in the metformin and ATO-induced antiproliferative effect on ICC cells, we demonstrated the upregulation of ERK3 in ICC cells treated with both metformin and ATO using a western blot assay (Fig. 3c, d). Phosphorylated ERK3 (detected by an anti-MAPK6 (phospho S189) antibody from Abcam (ab 74032)) was detected, and no significant variation was found when compared to total EKR3 (data not shown). Therefore, part of the following work focused on the total ERK3 variation in the mechanism studies.

### Combined treatment with metformin and ATO efficiently reduces ICC growth in an ICC xenograft model

**Fig. 4:**
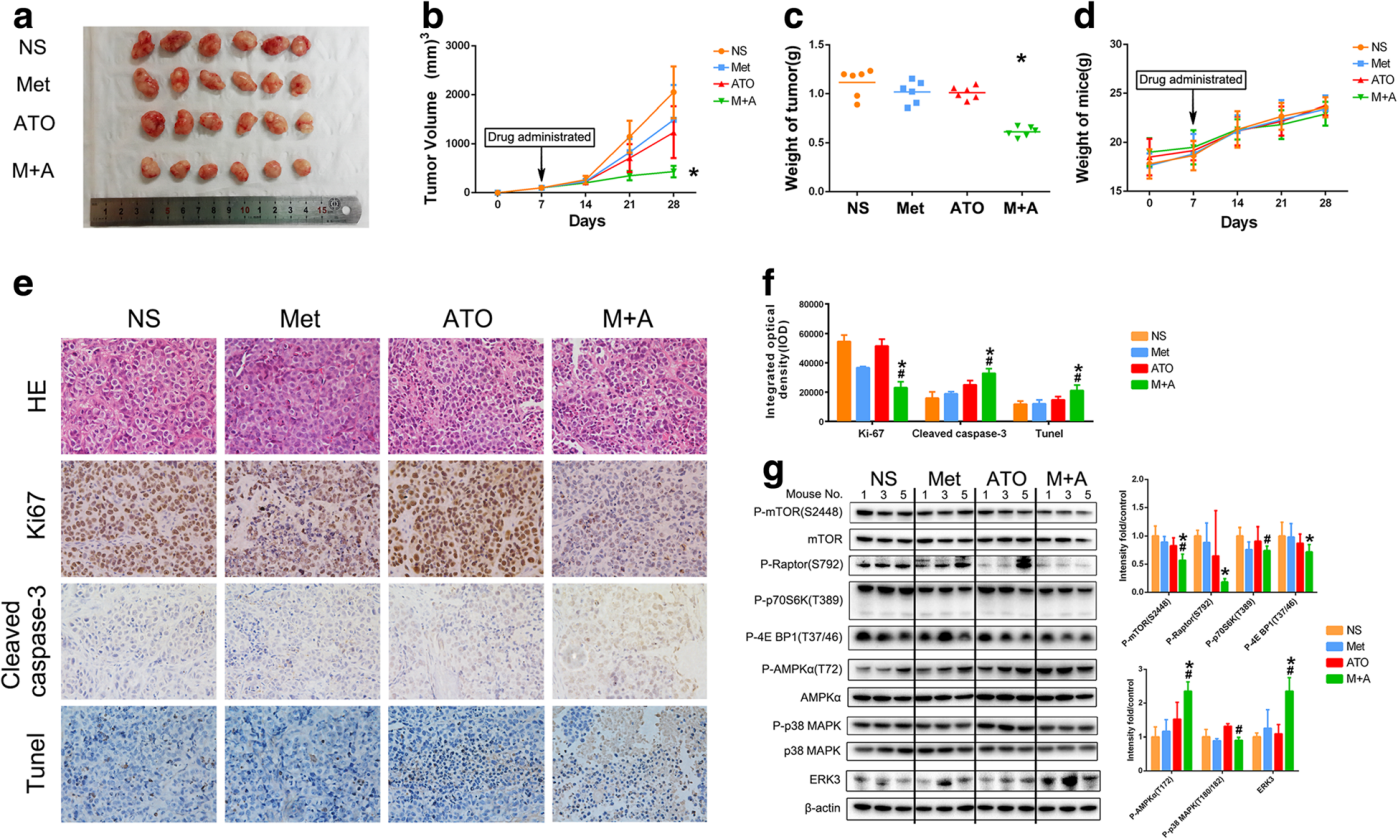
Metformin and ATO in combination potentiate the antiproliferative and pro-apoptotic effect of the single agent treatments in vivo through mTORC1, AMPK/MAPK, and ERK3 pathways. **a** CCLP-1 cells were implanted subcutaneously into the flank regions of nude mice. When the tumor volume reached approximately 100 mm^3^, vehicle (NS), metformin, ATO, or a combination of both were administered. (Combination vs metformin or combination vs ATO ^*^ *P* < 0.05). After 3 weeks, the mice were euthanized, and the tumors are shown. **b** and **c** Tumor volume and tumor weight were measured. **d** The weight of mice in each group was compared. **e** Representative hematoxylin-eosin (HE) stained images are shown, and the expression of Ki67 and cleaved caspase-3 in the tumors was detected by IHC. In addition, apoptotic cells in the xenografted tumors were detected by TUNEL assay. **f** The data were quantified and are presented as the means ± SD from three independent experiments. **g** The effect of metformin and ATO single or combination treatment on the active status of mTORC1, AMPK/MAPK, and ERK3 in xenografts was determined by western blotting. The data were quantified and are represented as the means ± SD. (Combination vs metformin ^*^ *P* < 0.05, combination vs ATO ^#^ *P* < 0.05)

To further validate our in vitro results showing the antiproliferative effects of combined treatment with metformin and ATO, we treated male athymic nude mice bearing palpable tumors (approximately 100 mm^3^) of CCLP-1 xenografts with control (vehicle-treated mice), metformin (200 mg/kg/day), ATO (3 mg/kg/day), and a combination of metformin and ATO (Figs. 4a–c) for 21 days. The tumor volumes of the combination treatment group were significantly reduced compared to the groups treated with either metformin or ATO alone. To evaluate the safety, the weights of the mice were obtained, and no significant variation was found (Fig. 4d), implying that treatment with a combination of metformin and ATO was very safe. Consistent with the in vitro data, immunohistochemistry and TUNEL analyses (Fig. 4e) of the xenograft tumors revealed that the metformin and ATO combination effectively inhibited the expression of Ki67, a marker representing tumor proliferation. Moreover, metformin and ATO promoted apoptosis in xenograft tumors, as evaluated by the cleaved caspase-3 staining and TUNEL assay.

### The combined treatment of metformin and ATO suppressed the active status of mTORC1 and regulated AMPK/p38 MAPK and ERK3 in ICC xenograft tumors, consistent with the results in vitro

Similarly, xenograft tumors were further examined for the status of mTORC1, AMPK/p38 MAPK, and ERK3 after treatment with the control, metformin, ATO, or a combination of both (Fig. 4f, g). Consistent with the in vitro results, metformin, and ATO cooperated to inactivate mTORC1, activate AMPK, and upregulate ERK3. Moreover, activation of p38 MAPK was also observed in mice in the ATO group, and this activation could be abrogated by metformin (Fig. 4f, g). These results supported the findings of the molecular network in the previous in vitro experiments.

### AMPK and p38 MAPK-dependence of the antiproliferative effect of metformin plus ATO on ICC cells

**Fig. 5:**
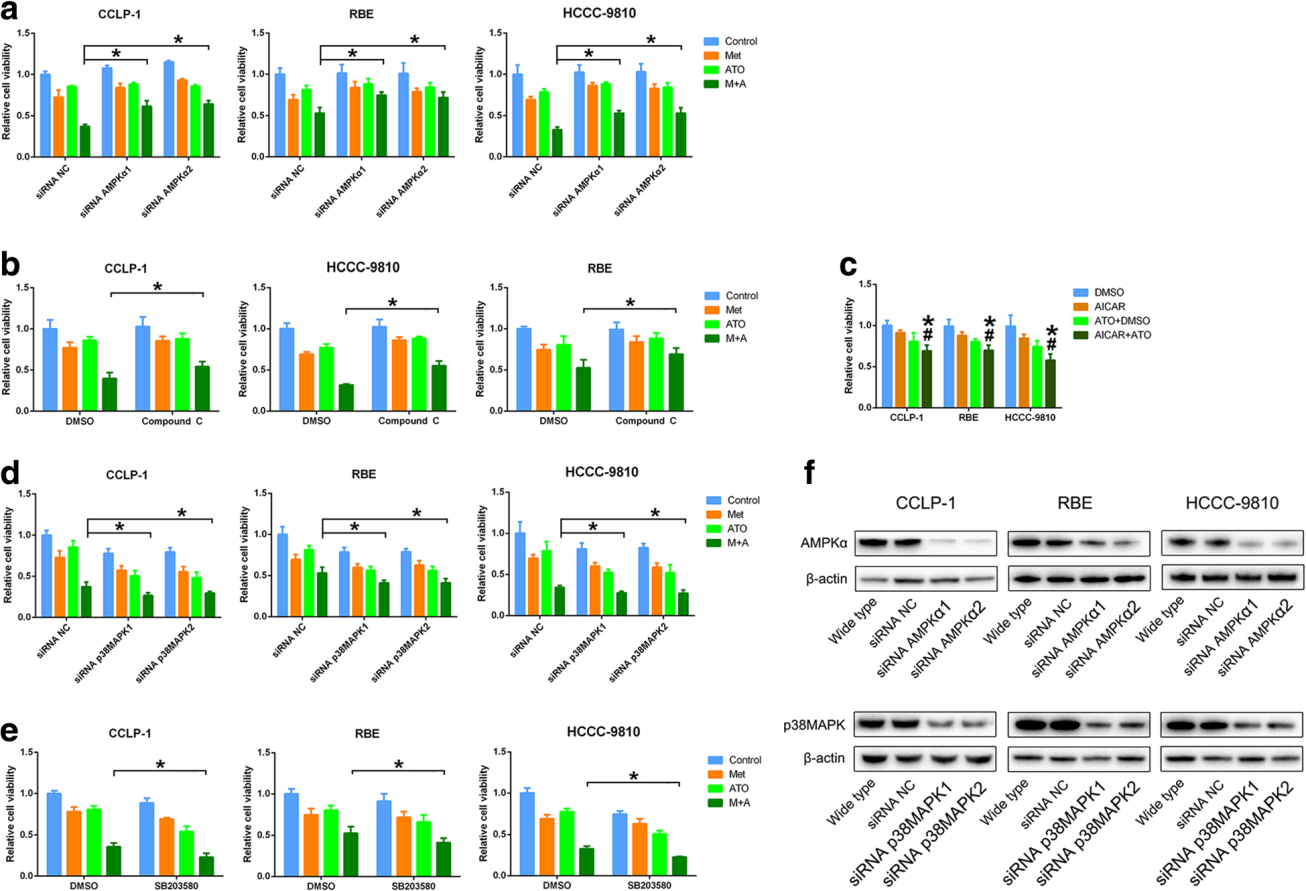
The role of AMPK and p38 MAPK in the antiproliferative effect of metformin and ATO combination treatment on ICC cells. **a** Downregulation of AMPK by specific siRNAs partially rescued ICC cell survival in cells treated with metformin and ATO. **b** Inactivation of AMPK by compound C partially rescued ICC cell survival in cells treated with metformin and ATO (^*^ *P* < 0.05). **c** The activator of AMPK, AICAR, potentiated the antiproliferative effect of ATO on ICC cells. (Combination vs AICAR ^*^ *P* < 0.05, combination vs ATO ^#^ *P* < 0.05). **d** and **e** Downregulation of p38 MAPK by specific siRNAs or inactivation of p38 MAPK by SB203580 potentiated the antiproliferative effect of metformin and ATO combination treatment on ICC cells. **f** The expression level of AMPK and p38 MAPK was detected by western blot in ICC cells after specific siRNA interference

To characterize the mechanism responsible for metformin-ATO synergism in ICC, we first thoroughly explored the roles of AMPK and p38 MAPK in the synergistic effect. Accordingly, pharmacological and genetic methods were used to regulate the expression level or active status of AMPK and p38 MAPK. As shown in Fig. 5a, b, and f, downregulation or inactivation of AMPK by short interfering RNA (siRNA) or by compound C [23] rescued the viability of ICC cells treated with the metformin and ATO combination. An AMPK activator, AICAR, significantly sensitized ICC cells to ATO (Fig. 5c), which further demonstrated the AMPK-dependence of the antiproliferative effect of metformin plus ATO on ICC cells. Additionally, similar methods were used for p38 MAPK, and the results (Fig. 5d–f) revealed that inactivation of p38 MAPK by SB203580 [24] or specific siRNAs promoted the effect of metformin and ATO on ICC cells, implying that metformin-induced abrogation of activated p38 MAPK by ATO may contribute to the metformin-ATO synergism in ICC cells.

### The role of ERK3 in the antiproliferative effect of metformin plus ATO on ICC cells

**Fig. 6:**
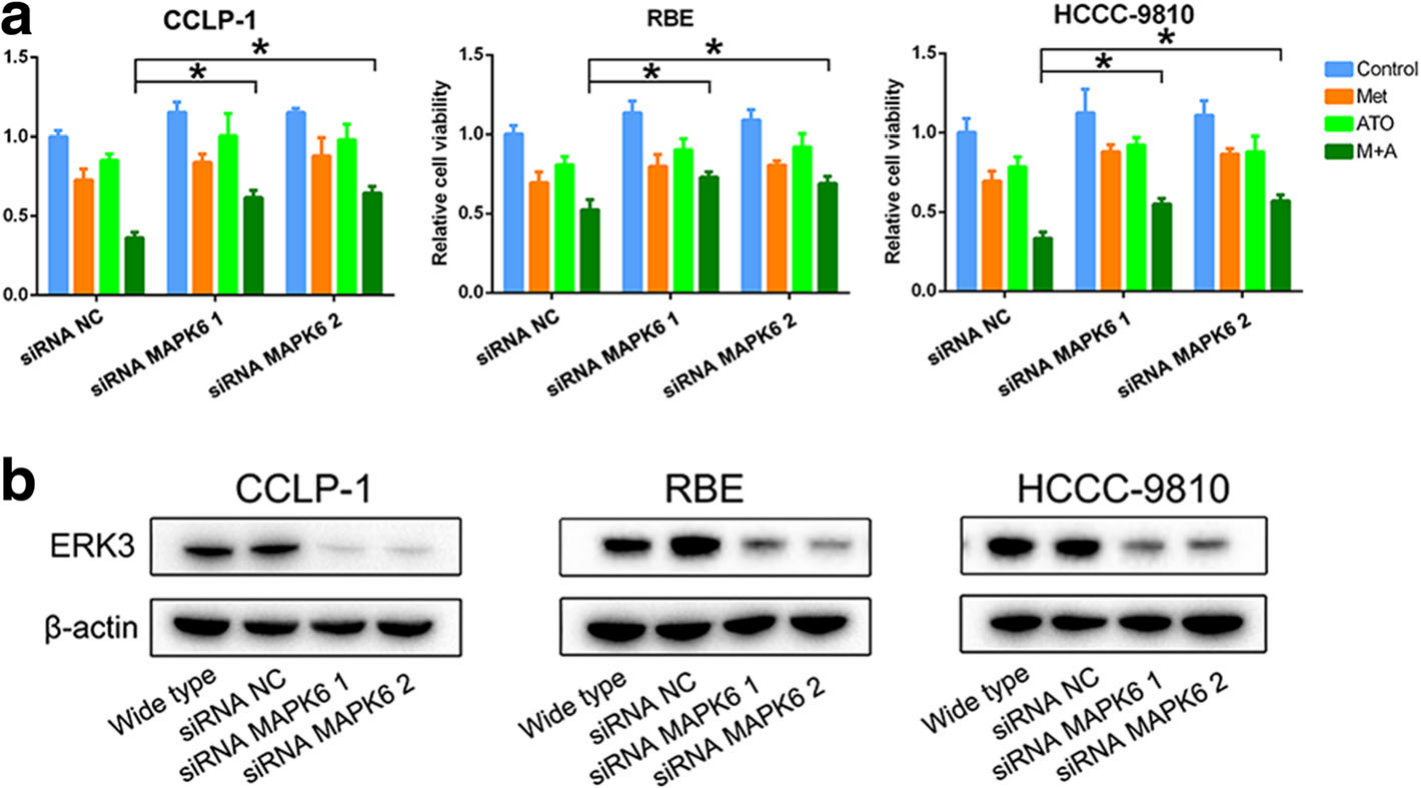
Downregulation of ERK3 rescues ICC cell survival in cells treated with both metformin and ATO. **a** Downregulation of AMPK by specific siRNAs partially rescued ICC cell survival in cells treated with metformin and ATO (^*^ *P* < 0.05). **b** The expression level of ERK3 was detected by western blot in ICC cells after MAPK6-specific siRNA interference

As shown in Fig. 6a, b, downregulation of ERK3 by MAPK6-specific siRNAs rescued the viability of ICC cells treated with metformin and ATO, which implied that ERK3 is a suppressive molecule in ICC cells.

### The regulation network among AMPK, p38 MAPK, ERK3, and mTORC1 in ICC cells

**Fig. 7:**
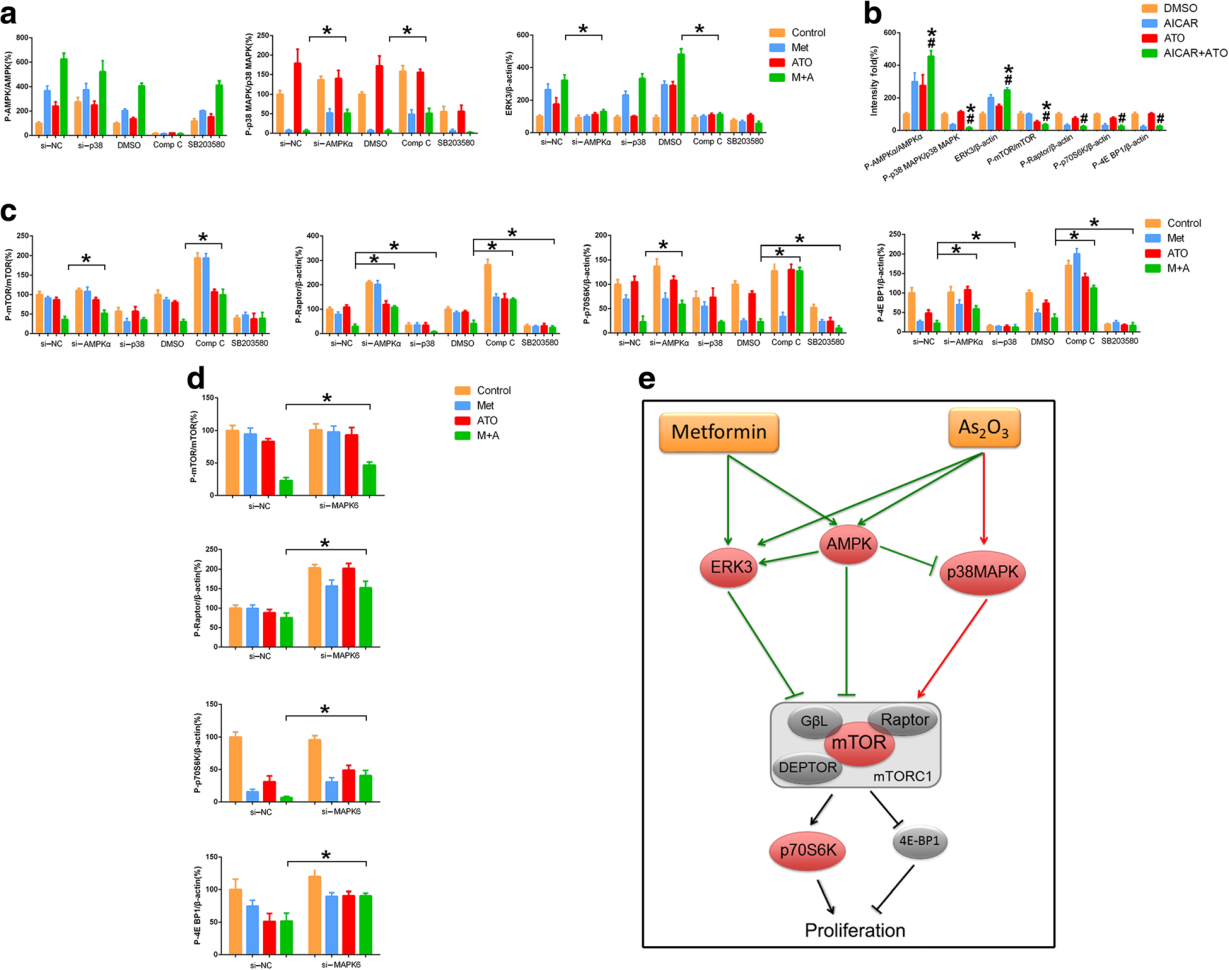
The role of AMPK, p38 MAPK, and ERK3 in metformin and ATO-induced mTORC1 inhibition in ICC cells. **a** The expression level of p-p38 MAPK/p38 MAPK and ERK3 was detected by western blot in ICC cells that were treated with metformin and ATO and in which AMPK or p-p38 MAPK was inhibited by siRNA or inhibitors (^*^ *P* < 0.05). **b** The activation status of AMPK, p38 MAPK, ERK3, and mTORC1 was detected by western blot in ICC cells treated with AICAR and ATO. (Combination vs AICAR ^*^ *P* < 0.05, combination vs ATO ^#^ *P* < 0.05). **c** The activation status of mTORC1 was detected by western blot in ICC cells that were treated with metformin and ATO and in which AMPK or p-p38 MAPK was inhibited by siRNA or inhibitors (^*^ *P* < 0.05). **d** The activation status of mTORC1 was detected by western blot in ICC cells treated with metformin and ATO and MAPK6-specific siRNA to downregulate ERK3 (^*^ *P* < 0.05). **e** The proposed relationship between AMPK, p38 MAPK, and ERK3 in response to metformin- and ATO-induced mTORC1 inhibition in ICC cells. The *green lines* indicate the inhibition of mTORC1 by metformin and ATO. The *red lines* indicate the activation of mTORC1 by ATO

Inactivation of AMPK by compound C or specific siRNA partially rescued metformin-induced inhibition of p38 MAPK and partially abrogated metformin plus ATO-induced upregulation of ERK3 and inhibition of mTORC1 in ICC cells (Fig. 7a, c). This finding was also supported by the molecular regulation observed in cells treated with AICAR and ATO, shown in Fig. 7b. For p38 MAPK, inactivation of p38 MAPK by SB203580 or specific siRNA promoted the inactivation of mTORC1 in ICC cells treated with metformin and ATO (Fig. 7c); however, our results did not reveal the relationship between p38 MAPK and ERK3. (Fig. 7a) Moreover, downregulation of ERK3 by siRNA rescued the active status of mTORC1 inhibited by the metformin and ATO combination treatment (Fig. 7b), which implied that ERK3 is a suppressor of mTORC1.

Collectively, we propose a potential molecular mechanism in which metformin and ATO inhibit ICC development via modulation of a network involving the AMPK, p38 MAPK, ERK3, and mTORC1 pathways (Fig. 7e). Specifically, metformin and ATO cooperated to inhibit mTORC1 through activation of AMPK and upregulation of ERK3. Meanwhile, metformin abrogated the activation of p38 MAPK induced by ATO, which partially depended on AMPK activation. The western blot data of the three ICC cell lines are shown in Additional file 3.

### ERK3 functions as a suppressor in ICC development

**Fig. 8:**
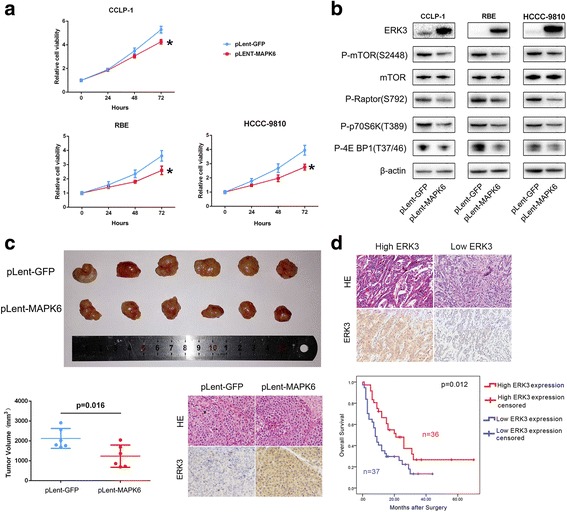
The biological and molecular roles of ERK3 in ICC. **a** ICC cells with lentiviral vector-mediated transfer of MAPK6 or GFP were assessed using a CCK-8 assay to evaluate the cell proliferation variation (^*^ *P* < 0.05). (**b**) The mTORC1 status in ICC cells with lentiviral vector-mediated transfer of MAPK6 or GFP was detected by western blot. **c** CCLP-1 cells with lentiviral vector-mediated transfer of MAPK6 or GFP were implanted subcutaneously into the flank regions of nude mice. After 4 weeks, the mice were euthanized, and the tumors are shown. The expression of ERK3 in the xenografts was detected by IHC. **d** ERK3 was detected by IHC in tumors from 73 ICC patients after surgery. The relevance of the ERK3 expression level and the prognosis of ICC patients was evaluated by the Kaplan-Meier method

The role of ERK3 in cancer is rarely explored. Therefore, we further explored the function of ERK3 in ICC. We found that overexpression of EKR3 in ICC cells inhibited cell proliferation (Fig. 8a) through inactivation of mTORC1 (Fig. 8b). Overexpression of EKR3 also inhibited the ICC xenograft growth (Fig. 8c). In addition, to explore whether ERK3 could be a promising molecular marker for predicting the prognosis of ICC patients, we detected the expression level of ERK3 in tumor samples from 73 ICC patients and compared the survival times of the patients with the expression level (high or low) of ERK3 (Additional file 4). As shown in Fig. 8d, high ERK3 expression is associated with a better prognosis in ICC patients. In addition, COX multivariate analysis showed that expression of ERK3 (low) and TMN stages (III and IV) are the independent risk factors for shorter overall survival times (Table 2 in Additional file 5). Collectively, these data implied that ERK3 is a suppressor of ICC development. Moreover, when correlated with clinical findings, we found that the expression level of ERK3, segregated as high or low, displayed a significant correlation with vessel invasion, implying that ERK3 has an antiangiogenic effect in ICC (Table 1 in Additional file 4), which is worth further exploration in future studies.

## Discussion

We demonstrated the synergistic interaction between metformin and ATO in ICC cells. Metformin significantly potentiates the effect of ATO against ICC in vitro and in vivo. This interaction facilitates ROS production, apoptosis, and cell cycle arrest. The PI3K-AKT-mTOR, Ras-MAPK, JAK-STAT, and Notch pathways are the primary signaling networks in cholangiocarcinoma [1, 25]. The PI3K-AKT-mTOR and Ras-MAPK pathways have profound effects on cell survival in cholangiocarcinoma [25], and they have been identified as potential therapeutic targets in cancer treatment [26, 27]. However, single-drug-based therapy strategies typically fail because of the crosstalk between the PI3K-AKT-mTOR and Ras-MAPK pathways [28, 29]. In the present study, we evaluated the status of AMPK/mTORC1 and MAPK underlying the synergistic antitumor effect of combined metformin and ATO treatment in ICC cells.

As we described in our previous study [6], whether activation of the mTOR pathway promotes the development of cholangiocarcinoma or predicts a poor prognosis remains controversial. However, mTORC1 has been demonstrated to be significant for the growth of cholangiocarcinoma [30]. A prospective pilot study reported that the mTORC1 inhibitor sirolimus can induce temporary partial remission or stabilization of cholangiocarcinoma [31]. Thus, we deduced that a combination of metformin and ATO would synergistically abrogate the activation of mTORC1 in ICC in vitro and in vivo, which may be partially be responsible for the synergistic anticancer effect of the two agents. The p38 MAPK pathway is a promising target of ATO. ATO activated p38 MAPK and subsequently inhibited the Akt/mTOR signaling pathways to suppress tumor cell growth [32, 33]. Conversely, p38 MAPK may promote tumorigenesis and development in cholangiocarcinoma [34, 35]. Thus, we evaluated the role of ATO-triggered activation of p38 MAPK in growth suppression of cholangiocarcinoma. Our results suggested that inactivation of p38 MAPK by the p38 MAPK inhibitor SB203580 or p38 MAPK-specific siRNA could enhance the anticancer effect of single agent or combined metformin and ATO treatment, especially the ATO single-drug treatment. Thus, activation of p38 MAPK may be responsible for resistance to ATO in cholangiocarcinoma. These results are consistent with some other finding regarding the MAPK-induced resistance of cancer cells to ATO [36]. Metformin significantly inhibited the ATO-induced activation of p38 MAPK in ICC cells in vitro and in vivo, which may also mechanistically explain the synergistic anticancer effect of the two agents.

ERK3, encoded by MAPK6, is an atypical member of the MAPK family, which can suppress cell proliferation via the ERK3-MAPK-activated protein kinase-5(MK5) pathway [19]. In our study, metformin and ATO increased the expression of ERK3 via activation of AMPK. ERK3 partially bridged the inactivation of mTORC1 induced by metformin and ATO in ICC cells. Moreover, we are the first to report that increased expression of ERK3 inhibited ICC cell growth in vitro and in vivo, and high ERK3 expression in tumor samples predicted a better prognosis in ICC patients after tumor resection. Therefore, ERK3 may be a protective molecule in ICC progression. The functions of ERK3 are poorly understood. MK5 is a recognized downstream target of ERK3, which can phosphorylate MK5 on Thr-182 [19]. The phosphorylation activity of MK5 may be responsible for energy depletion-induced suppression of mTORC1 [37]. In the present study, we demonstrated that ERK3 is a suppressor of mTORC1, which we speculate may be mediated by activation of MK5. For future studies, we should further investigate the role of the ERK3-MK5-mTORC1 pathway in the antitumor effect of combined metformin and ATO treatment in ICC cells, which may be the primary deficiency of our study. p38 MAPK is another potential activator of MK5 [37, 38]. Our results did not suggest that regulation between p38 MAPK and ERK3 occurs. However, as we described previously, inactivation of p38 MAPK mechanistically bridged the synergistic anticancer effect of metformin and ATO. Therefore, the p38 MAPK-MK5 pathway is also worth exploring in detail. Furthermore, investigation of the influence of ERK3 on biological activity, such as apoptosis and drug sensitivity in ICC cells, is needed.

In regard to the regulation of p38 MAPK and ERK3, few studies have reported an AMPK-dependent effect. As a significant target of metformin, AMPK bridged the primary activity of metformin [23]. Activation of AMPK by metformin or AICAR activated p38 MAPK and increased the expression of ERK3. Nevertheless, we did not find that the activation of p38 MAPK by ATO was influenced by modulation of AMPK, which may be explained by the fact that direct activation of p38 MAPK by ATO overcomes the inhibitory effect of ATO-triggered AMPK activation. Moreover, for EKR3, AMPK is a newfound positive regulator, although more mechanisms are likely to be involved in metformin and ATO-triggered increased expression of ERK3.

## Conclusions

Metformin potentiates the effect of arsenic trioxide suppressing ICC cells in vitro and in vivo. Because metformin or ATO alone does not seem to be beneficial for ICC patients or patients with certain other solid tumors, treatment of ICC patients with a combination of metformin and ATO-based chemotherapy could be considered in a clinical setting. Mechanistically, we demonstrated that metformin and ATO targeted both the PI3K-AKT-mTOR and Ras-MAPK pathways, the crosstalk of which is disrupted by metformin and ATO through AMPK/p38 MAPK-ERK3/mTORC1 pathway. ERK3 is a newfound molecular marker for predicting the prognosis of ICC patients after resection, and the function of ERK3 in ICC needs to be investigated in future studies. Detection of the p38 MAPK activation status and the expression of ERK3 are valuable for evaluating the sensitivity of ICC to metformin and ATO.

## Methods

### Cell culture

Three ICC cell lines, CCLP-1 [39], RBE, and HCCC-9810, were used. The CCLP-1 cell line was obtained from DSMZ (Braunschweig, Germany). RBE and HCCC-9810 cells were purchased from the Type Culture Collection of the Chinese Academy of Sciences (Shanghai, China). CCLP-1 cells were cultured in Dulbecco’s modified Eagle’s medium (DMEM)-high glucose (Gibco, USA). RBE and HCCC-9810 cells were cultured in RPMI-1640 (Gibco, USA) supplemented with 10% fetal bovine serum (FBS; Gibco) and 100 μg/ml each of penicillin and streptomycin (Invitrogen, USA) in 5% CO_2_ at 37°°C.

### Reagents

Metformin (1,1-dimethylbiguanide hydrochloride, #D150959-5G) and AICAR(5-Aminoimidazole-4-carboxamide 1-β-D-ribofuranoside Acadesine N1-(β-D-Ribofuranosyl)-5-aminoimidazole-4-carboxamide, A9978) were purchased from Sigma-Aldrich (St. Louis, MO, USA). Compound C (Dorsomorphin, 6-[4-(2-Piperidin-1-ylethoxy)phenyl]-3-pyridin-4-ylpyrazolo[1,5-a]pyrimidine, P5499) and SB239063 (trans-1-(4-Hydroxycyclohexyl)-4-(4-fluorophenyl)-5-(2-methoxypyridimidin-4-yl)imidazole, S7741) were purchased from Selleck Chemicals (Houston, TX, USA). Arsenic trioxide (As2O3, ATO) was purchased from Shuanglu Pharmaceutical Co., Ltd. (Beijing, China). The cell counting kit-8 (CCK-8, KGA317), the Annexin V-FITC Apoptosis Detection Kit (KGA108), the cell cycle detection kit (KGA512), Caspase3/7 Activity Detection Kit (KGAS037), the terminal deoxynucleotidyltransferase-mediated deoxyuridine triphosphate (dUTP) nick-end labeling (TUNEL) Assay Kit (KGA707), and the ROS (reactive oxygen species) Detection Kit (KGT010) were purchased from KeyGen Biotech (Nanjing, China). The HistostainTM-Plus Kits (IgG/Bio, S-A/HRP, DAB) were purchased from Zhongshan Golden Bridge Co., Ltd. (Beijing, China).

### Antibodies

The following antibodies were used for immunoblotting or immunohistochemical staining: β-actin (sc-47778) was from Santa Cruz Biotechnology, Inc. Sant (Santa Cruz, CA, USA). AMPKα (#2532) and phosphorylated AMPKα (Phospho-Thr172, #2535), mTOR (#2983), phosphorylated mTOR (Phospho-Ser2448, #5536), phosphorylated Raptor (Phospho-Ser792, #2083), phosphorylated p70 S6 kinase (Phospho-Thr389, #9234), phosphorylated 4E-BP1(Phospho-Thr37/46, #2855), cleaved PARP (#5626), cleaved caspase-3(#9661), ERK (#4696), phosphorylated ERK(Phospho-Thr202/Tyr204, #4370), p38 MAPK(#8690), phosphorylated p38 MAPK(Thr180/Tyr182, #4511), and Ki-67 (#9449) were from Cell Signaling Technology, Inc. (Danvers, MA, USA). ERK3(ab53277) was from Abcam (Cambridge, MA, USA). Goat anti-rabbit and goat anti-mouse IgG peroxidase-conjugated secondary antibodies (31460 and 31430) were from Thermo-Pierce (Rockford, IL, USA).

### Real-time cell growth monitoring

The real-time cell growth was monitored using a 16 × E-Plates RTCA DP Analyzer (ACEA Biosciences). After 5000 cells were seeded in a well of 16 × E-Plates for 16 to 24 h, the medium was replaced by fresh medium with certain agents (control, metformin (10 mmol/L), ATO (3 μmol/L) or a combination of both). Then, the cell index, representing the cell viability, was detected every 15 min for 72 h. Four replicates were prepared for each condition. The growth cures are shown.

### Cell viability assay

Cell viability was determined using a CCK-8 assay according to the manufacturer’s instructions. The detailed procedure has been described previously [6].

### Determination of synergism

The synergism of metformin and ATO was determined using the method of Chou [17] and the software package Calcusyn (Biosoft, Cambridge, United Kingdom). Combination indices (CI) were calculated, and a CI less than 1 was defined as synergism.

### Cell cycle, apoptosis, and ROS evaluation

The cell cycle arrest, induction of apoptosis, and intracellular ROS levels were determined by flow cytometry. Portions of the detailed procedure have been described previously [6].

For the detection of intracellular ROS, an oxidation-sensitive fluorescent probe (DCFH-DA) was used. After treatment with agents for 24 h, a total of 1 × 10^6^ cells were trypsinized and pelleted by centrifugation and washed twice with PBS. Then, the cell pellets were resuspended in 1 mL of DMEM or RPMI-1640 (serum-free) with 10 μm/L DCFH-DA and incubated for 20 min at 37 °C. The stained cells were analyzed using a flow cytometer (Accuri Cytometers Inc.). The FL1-A received the fluorescence induced by DCF. For each sample, 10, 000 events were collected.

A Caspase3/7 activity detection kit was used according to the manufacturer’s instructions. Briefly, after treatment with agents for 48 h, 1 × 10^6^ cells were incubated in 1 μm/L working solution for 30 min at 37 °C. Finally, the optical density was measured using a microplate reader (Thermo, Varioskan Flash).

### Phospho-antibody array

Phosphoprotein profiling by the cancer-signaling phospho-antibody microarray—the cancer-signaling phospho-antibody microarray PCS248, which was designed and manufactured by Full Moon Biosystems, Inc. (Sunnyvale, CA), contains 248 antibodies. Each of the antibodies has six replicates that are printed on coated glass microscope slides, along with multiple positive and negative controls. The antibody array experiment was performed by Wayen Biotechnology (Shanghai, China), according to their established protocol. Briefly, cell lysates obtained from CCLP-1 cells treated with single or double agents were biotinylated with an Antibody Array Assay Kit (Full Moon Biosystems, Inc.). The antibody microarray slides were first blocked in a blocking solution (Full Moon Biosystems, Inc.) for 30 min at room temperature, rinsed with Milli-Q grade water for 3–5 min, and dried with compressed nitrogen. The slides were then incubated with the biotin-labeled cell lysates (~100 μg of protein) in coupling solution (Full Moon Biosystems, Inc.) at room temperature for 2 h. The array slides were washed 4–5 times with 1X Wash Solution (Full Moon Biosystems, Inc.) and rinsed extensively with Milli-Q grade water before detection of bound biotinylated proteins using Cy3-conjugated streptavidin. The slides were scanned on a GenePix 4000 scanner, and the images were analyzed with GenePix Pro 6.0 (Molecular Devices, Sunnyvale, CA). The fluorescence signal (I) of each antibody was obtained from the fluorescence intensity of antibody-stained regions. A ratio computation was used to measure the extent of protein phosphorylation. The phosphorylation ratio was calculated as follows: phosphorylation ratio = phospho value/non-phospho value. The total proteome ratios were standardized by β-actin.

### Western blot analysis

Cells after different treatments or tumor tissues from a xenograft were harvested for western blot analysis. The detailed procedure has been described previously [6]. Primary antibodies (described in the Antibodies section) were incubated at 4 °C overnight. The bands were visualized by chemiluminescence, imaged using a ChemiDoc XRS and analyzed using Image Lab (both from Bio-Rad).

### Xenograft model analysis

To investigate the antiproliferative effect of metformin and ATO in combination treatment on ICC cells in vivo, a model of nude mice bearing ICC cell xenografts was established. Five-week-old male athymic nude mice were obtained from the Animal Facility of Zhejiang University. The mice were maintained under pathogen-free conditions and were provided with sterilized food and water. Briefly, 5 × 10^6^ CCLP-1 cells were injected subcutaneously into the right flank of each nude mouse. When mice exhibited palpable tumors (the tumor volume was approximately 100 mm^3^), they were randomly divided into control (100 μL of NS by intraperitoneal injection), metformin (150 mg/kg/day diluted in 100 μL of NS by intraperitoneal injection), ATO (2.5 mg/kg/day diluted in 100 μL of NS by intragastric administration), and a combination of both (metformin, 150 mg/kg/day plus ATO 3 mg/kg/day diluted in 100 μL of NS by intraperitoneal injection) groups (*n* = 6 animals per group). The treatments were performed five times per week for 3 weeks. The tumor volume was detected every week and was calculated using the following formula: volume = 1/2 (length × width^2^). After 4 weeks, all the mice were sacrificed, and the tumors were isolated. NS means normal saline.

To evaluate the antiproliferative effect of ERK3 in ICC in vivo, 5 × 10^6^ CCLP-1 cells with ERK3 overexpression or GFP transfection were injected subcutaneously into the right flank of each nude mouse. After 4 weeks, the tumor volume was determined and calculated, as previously described, all the mice were sacrificed, and the tumors were isolated.

### Immunohistochemical staining and evaluation

The tumors isolated from the mice were paraffin embedded and cut into 10-μm-thick sections in a microtome cryostat (HM500 OM, Carl Zeiss, Germany). Immunohistochemical staining was conducted according to the manufacturer protocols described for HistostainTM-Plus Kits. Primary antibodies (described in the Antibodies section) were incubated at 4 °C overnight. Images were captured with a light microscope (Axiolab, Carl Zeiss, Germany), and five images/sample were prepared. Image-Pro Plus 4.5 Software was used to analyze the staining data.

ERK3 expression in the 73 cases was evaluated by two of the authors (Hai-Yang Xie and Fan Yang), who were blind to the clinicopathological data. Any discrepancy between the two individuals was resolved by a third individual. A semiquantitative scoring system was used. Brown granules in the cytoplasm representing ERK3 were considered positive staining. We scored the staining intensity as follows: 0, no staining; 1+, mild staining; 2+, moderate staining; 3+, intense staining. The area of staining was evaluated as follows: 0, no staining of cells in any microscopic field; 1+, <30% of tissue stained positive; 2+, between 30 and 60% of tissue stained positive; and 3+, >60% of tissue stained positive. ERK3 expression was evaluated by combining the staining intensity and area assessments. The minimum score when summed (intensity + extension) was 0, and the maximum score was 6. Scores ≤3 were defined as low expression, and scores >3 were defined as high expression.

### TUNEL assay

In situ detection of apoptotic cells in the tumors isolated from the mice was performed with a TUNEL assay. The tumors were paraffin embedded and cut into 10-μm-thick sections in a microtome cryostat (HM500 OM, Carl Zeiss, Germany). The TUNEL assay was conducted according to the manufacturer’s protocols. 3,3-Di- aminobenzidine (DAB) was used as the substrate for the peroxidase. Images were captured with a light microscope (Axiolab, Carl Zeiss, Germany), and five images/sample were prepared. Image-Pro Plus 4.5 Software was used to analyze the staining data.

### Gene knock-down using siRNA

ICC cells were seeded in a 6-well plate at a concentration of 2 × 10^5^ cells per 2 ml in medium with FBS, penicillin, and streptomycin. After 24 h, the medium was replaced with fresh medium without antibiotics or FBS. Meanwhile, siRNA or a negative control oligonucleotide was transfected into the cells with Lipofectamine 3000, according to the manufacturer’s instructions. After 24 h, the medium was replaced with fresh complete medium. Then, the cells were cultured for subsequent experiments. All siRNAs were obtained from Shanghai GenePharma Co., Ltd. China; the sequences of siRNAs are listed in Additional file 6.

### Stable overexpression of ERK3 (encoded by MAPK6) in ICC cells

Lentiviral particles containing MAPK6 vector or empty vector were obtained from Shandong Vigenebio Co., Ltd. (Jinan, China). Cells were plated in a six-well plate at 2 × 10^5^ cells/well in supplemented medium 24 h before viral infection. Media were removed from well plates and replaced with media supplemented with Polybrene at a final concentration of 5 μg/ml. Next, cells were infected by adding lentiviral particles to the culture. Stable clones expressing MAPK6 or GFP were selected using puromycin dihydrochloride. Then, cells were collected for gene expression assays.

### Patient samples

Tumor samples from a total of 73 ICC patients who underwent operations at our hospital (First Affiliated Hospital, Zhejiang University School of Medicine, Zhejiang, China) between 2010 and 2014 were used in the present study. These patients were diagnosed with ICC either before or after surgery. No treatment was done for the patients before surgery. The diagnosis was confirmed by histopathological examination, and complete clinical and laboratory data were collected before surgery and during follow-up. The distribution of clinicopathological data in the study cohort is given in Table 1 of Additional file 4. Specimens of cancer tissues and clinical information were available from these patients after obtaining informed consent.

### Statistical analysis

SPSS 21.0 statistical software was used for the statistical analysis. Values are presented as the mean ± SD. Statistical analyses were performed using Student’s *t*-test. The analysis of multiple groups was performed by ANOVA with an appropriate post hoc test.

## Additional files

Additional file 1: The concentrations of metformin(Met) and arsenic trioxide(ATO) in each group and its corresponding combined antiproliferative effect values and CI. (DOCX 14 kb)
Additional file 2: Proteome and phosphoproteome array data. (TIF 966 kb)
Additional file 3: The western blot data of the three ICC cell lines. (TIF 9443 kb)
Additional file 4: Clinicopathological correlation of ERK3 expression in 73 ICC patients. (DOCX 14 kb)
Additional file 5: Univariate and multivariate survival analyses of potential predictors of overall survival in ICC patients following operation. (DOCX 17 kb)
Additional file 6: The sequences of siRNAs used in this study. (DOCX 36 kb)

## Abbreviations

AMPK: AMP-activated protein kinase
APL: Acute promyelocytic leukemia
ATO: Arsenic trioxide
CI: Combination index
ERK: Extracellular signal-regulated kinases
HCC: Hepatocellular carcinoma
ICC: Intrahepatic cholangiocarcinoma
MAPK: Mitogen-activated protein kinase
MK5: MAPK-activated protein kinase-5
mTORC1: mTOR complex 1
TACE: Transarterial chemoembolization
