# Supplementary material for: Metformin potentiates the effect of arsenic trioxide suppressing intrahepatic cholangiocarcinoma: roles of p38 MAPK, ERK3, and mTORC1
Source: J Hematol Oncol. 2017 Feb 28;10:59. doi: 10.1186/s13045-017-0424-0 (PMC5329912; doi:10.1186/s13045-017-0424-0)
Supplement: Additional file 1: — The concentrations of metformin(Met) and arsenic trioxide(ATO) in each group and its corresponding combined antiproliferative effect values and CI. (DOCX 14 kb) [file 13045_2017_424_MOESM1_ESM.docx]

Supplement file 1

The concentrations of metformin(Met) and arsenic trioxide(ATO) in each group and its corresponding combined anti-proliferative effect values and CI

|  | CCLP-1 | | RBE | | HCCC-9810 | |
| --- | --- | --- | --- | --- | --- | --- |
| Met(mM)/ATO(μM) | Anti-proliferative effect | CI | Anti-proliferative effect | CI | Anti-proliferative effect | CI |
| 2.5/1.5 | 0.159 | 1.1837 | 0.2318 | 0.6641 | 0.149 | 1.2037 |
| 2.5/3 | 0.416 | 0.8383 | 0.3824 | 0.8077 | 0.302613159 | 0.98762 |
| 5/3 | 0.6536 | 0.72596 | 0.4806 | 0.8809 | 0.6734951 | 0.97879 |
| 10/3 | 0.7603 | 0.7439 | 0.5196 | 0.9045 | 0.562062529 | 0.98824 |
| 5/6 | 0.8392 | 0.76029 | 0.6044 | 0.9069 | 0.869640691 | 0.66568 |
| 10/6 | 0.912 | 0.64486 | 0.7023 | 0.9143 | 0.911105926 | 0.64556 |
