# Supplementary figures and images for: Metformin potentiates the effect of arsenic trioxide suppressing intrahepatic cholangiocarcinoma: roles of p38 MAPK, ERK3, and mTORC1
Source: J Hematol Oncol. 2017 Feb 28;10:59. doi: 10.1186/s13045-017-0424-0 (PMC5329912; doi:10.1186/s13045-017-0424-0)

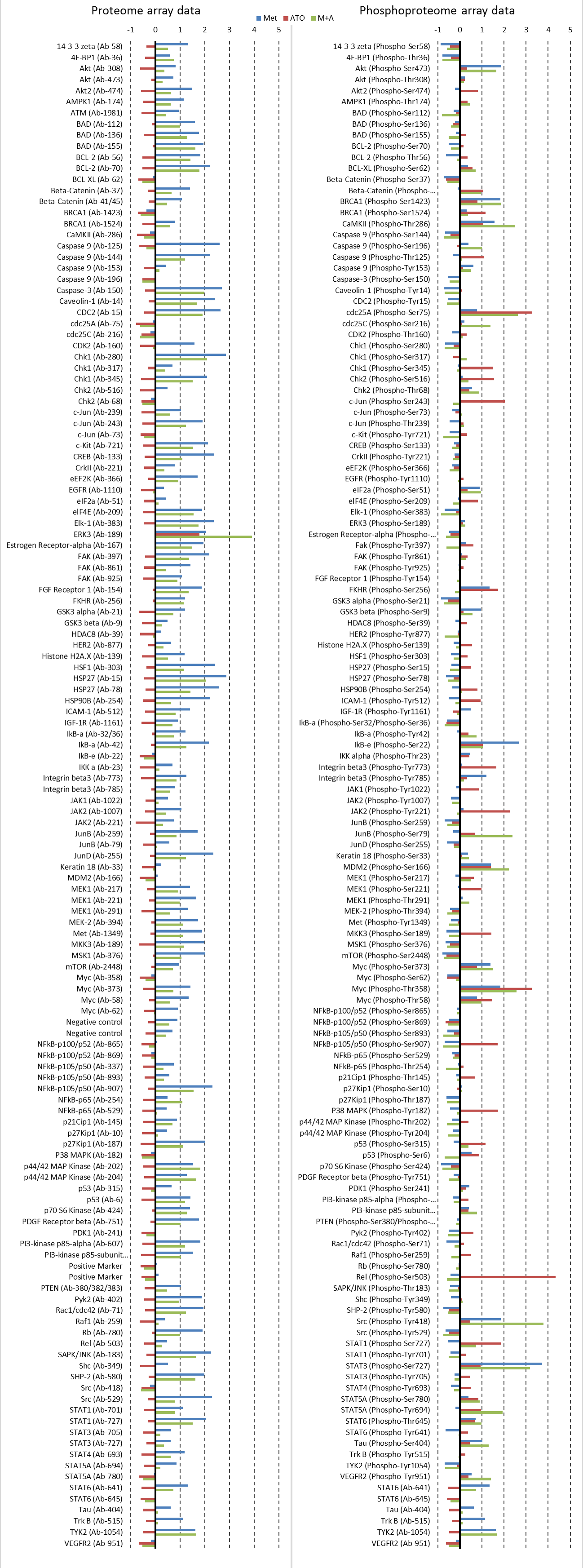

Supplement: Additional file 2: — Proteome and phosphoproteome array data. (TIF 966 kb) [file 13045_2017_424_MOESM2_ESM.tif]

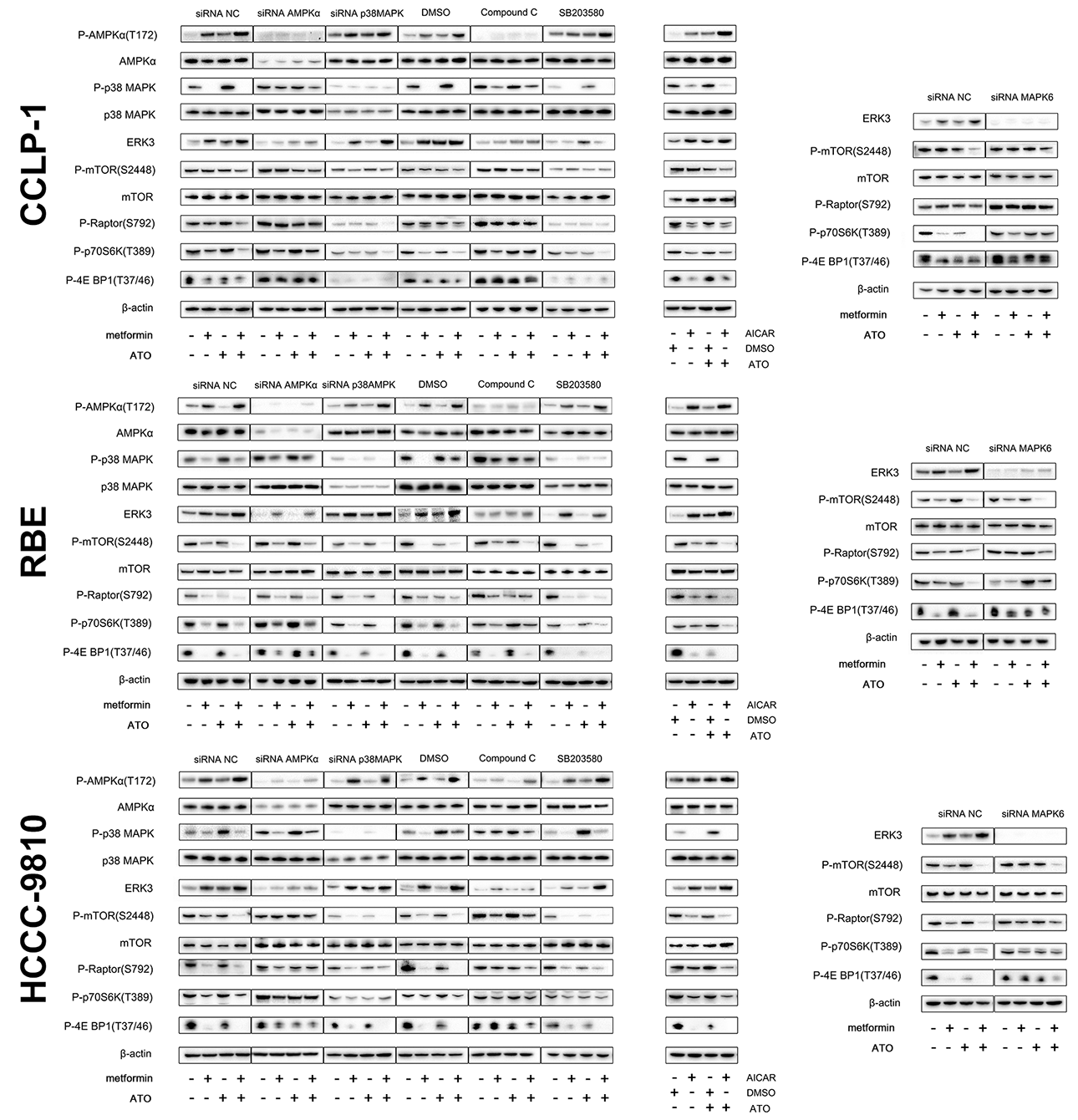

Supplement: Additional file 3: — The western blot data of the three ICC cell lines. (TIF 9443 kb) [file 13045_2017_424_MOESM3_ESM.tif]
