# Supplementary material for: Metformin potentiates the effect of arsenic trioxide suppressing intrahepatic cholangiocarcinoma: roles of p38 MAPK, ERK3, and mTORC1
Source: J Hematol Oncol. 2017 Feb 28;10:59. doi: 10.1186/s13045-017-0424-0 (PMC5329912; doi:10.1186/s13045-017-0424-0)
Supplement: Additional file 4: — Clinicopathological correlation of ERK3 expression in 73 ICC patients. (DOCX 14 kb) [file 13045_2017_424_MOESM4_ESM.docx]

**TABLE 1 Clinicopathological correlation of ERK3 expression in 73 ICC patients**

| Variables | Tumor ERK3 expression | | *P* value^a^ |
| --- | --- | --- | --- |
|  | Low(37) | High(36) |  |
| Age |  |  |  |
| ≤60 years | 12 | 16 | .291 |
| >60 years | 25 | 20 |  |
| Gender |  |  |  |
| Male | 20 | 20 | .897 |
| Female | 17 | 16 |  |
| Preoperative CA19-9 level |  |  |  |
| ≤37 kU/L | 11 | 5 | .102 |
| >37 kU/L | 26 | 31 |  |
| Histopathologic grading |  |  |  |
| Well + moderately | 14 | 18 | .295 |
| Poorly | 23 | 18 |  |
| Tumor size |  |  |  |
| ≤5 cm | 13 | 9 | .345 |
| >5 cm | 24 | 27 |  |
| TNM staging |  |  |  |
| Ⅰ+Ⅱ | 19 | 23 | .279 |
| Ⅲ+Ⅳ | 18 | 13 |  |
| Vascular invasion |  |  |  |
| Negative | 28 | 34 | .025 |
| Positive | 9 | 2 |  |

^a^ Statistical analyses were performed with chi-square test.
