# Supplementary material for: Metformin potentiates the effect of arsenic trioxide suppressing intrahepatic cholangiocarcinoma: roles of p38 MAPK, ERK3, and mTORC1
Source: J Hematol Oncol. 2017 Feb 28;10:59. doi: 10.1186/s13045-017-0424-0 (PMC5329912; doi:10.1186/s13045-017-0424-0)
Supplement: Additional file 5: — Univariate and multivariate survival analyses of potential predictors of overall survival in ICC patients following operation. (DOCX 17 kb) [file 13045_2017_424_MOESM5_ESM.docx]

**TABLE 2 Univariate and multivariate survival analyses of potential predictors of overall survival in ICC patients following operation**

| Variables | | Univariate analysis | | | | | *P* value^a^ | | Multivariate analysis | *P* value^b^ | |  |
| --- | --- | --- | --- | --- | --- | --- | --- | --- | --- | --- | --- | --- |
|  |  | HR 95% CI | | | | |  |  | HR 95%CI |  |  |  |
| Age | |  | | | | |  | |  | | |  |
| >60 years VS ≤60 years | | 1.774 | | 0.988-3.188 | | | 0.055 | |  | | | |
| Gender | |  | | | | |  | |  | | |  |
| Male VS Female | | 1.268 | | | | 0.731-2.201 | 0.389 | |  | | |  |
| Preoperative CA19-9 level | |  | | | | |  | |  | | |  |
| >37 kU/L VS ≤37 kU/L | | 1.564 | | | | 0.761-3.213 | 0.224 | |  | | |  |
| Histopathologic grading | |  | | | | |  | |  | | |  |
| Poorly VS Well + moderately | | 0.863 | | | | 0.500-1.489 | 0.597 | |  | | |  |
| Tumor size | |  | | | | |  | |  | | |  |
| >5 cm VS ≤5 cm | | 0.594 | | | 0.337-1.046 | | 0.071 | |  | | |  |
| TNM staging | |  | | | | |  | |  | | |  |
| Ⅲ+Ⅳ VS Ⅰ+Ⅱ | 2.021 | | 1.150-3.522 | | | | 0.014 | 1.791 1.016-3.157 | | | 0.044 |  |
| Vascular invasion | |  | | | | |  | |  | | |  |
| Positive VS Negative | | 0.766 | | | | 0.327-1.798 | 0.766 | |  | | |  |
| ERK3 expression | | | | | | | | | | | |  |
| Low VS High | | 1.980 | | | 1.139-3.448 | | 0.015 | | 1.783 1.014-3.134 | | 0.044 |  |

^a^ Statistical analyses were conducted by Kaplan–Meier method (logrank test)

^b^ Statistical analyses were conducted by Cox proportional hazards regression
