# Supplementary material for: Metformin potentiates the effect of arsenic trioxide suppressing intrahepatic cholangiocarcinoma: roles of p38 MAPK, ERK3, and mTORC1
Source: J Hematol Oncol. 2017 Feb 28;10:59. doi: 10.1186/s13045-017-0424-0 (PMC5329912; doi:10.1186/s13045-017-0424-0)
Supplement: Additional file 6: — The sequences of siRNAs used in this study. (DOCX 36 kb) [file 13045_2017_424_MOESM6_ESM.docx]

The sequences of siRNAs used in this study. (sense 5'-3')

MAPK6 1 GGCUUUUCAUGUAUCAGCU

MAPK6 2 GGAGUACAUGGAGACAGAC

AMPKα 1 GGAUCCAUCAUAUAGUUCA

AMPKα 2 CGUCAUUGAUGAUGAGGCU

p38 MAPK(MAPK14) 1 GGGCAGAUCUGAACAACAU

p38 MAPK(MAPK14) 2 AUGUUGUUCAGAUCUGCCC
